# Cytotoxicity of Silver Nanoparticle and Chitin-Nanofiber Sheet Composites Caused by Oxidative Stress

**DOI:** 10.3390/nano6100189

**Published:** 2016-10-20

**Authors:** Jun Kinoda, Masayuki Ishihara, Hidemi Hattori, Shingo Nakamura, Koichi Fukuda, Hidetaka Yokoe

**Affiliations:** 1 Department of Oral and Maxillofacial Surgery, National Defense Medical College, 3-2 Namiki, Tokorozawa, Saitama 359-8513, Japan; drom636@ndmc.ac.jp (J.K.); yokoe@ndmc.ac.jp (H.Y.); 2 Research Institute, National Defense Medical College, 3-2 Namiki, Tokorozawa, Saitama 359-8513, Japan; snaka@ndmc.ac.jp (S.N.); res309@ndmc.ac.jp (K.F.); 3 Department of Biochemistry and Applied Biosciences, University of Miyazaki, Miyazaki 889-2192, Japan; h-hattori@cc.miyazaki-u.ac.jp

**Keywords:** silver nanoparticles, chitin-nanofiber sheets, wound dressing, cytotoxicity, oxidative stress

## Abstract

Size-controlled spherical silver nanoparticles (<10 nm) and chitin-nanofiber sheet composites (Ag NPs/CNFS) have previously been reported to have strong antimicrobial activity in vitro. Although Ag NPs/CNFS have strong antimicrobial activity, their cytotoxicity has not been investigated. This study was performed to evaluate the effects of Ag NPs/CNFS on cytotoxicity for fibroblasts in vitro and healing delay of wound repair in vivo, focused on oxidative stress. Cytotoxic activities of Ag NPs/CNFS were investigated using a fibroblast cell proliferation assay, nitric oxide/nitrogen dioxide (NO/NO_2_) measurement of the cell lysates in vitro, inhibitory effects of Ag NPs/CNFS on healing-impaired wound repair using diabetic mice in vivo, 8-hydroxy-2′-deoxyguanosine (8-OHdG) immunohistochemical staining of the skin sections, and generation of carbonyl protein in the wound was performed to evaluate cytotoxicity with oxidative stress. Ag NPs/CNFS exhibited cytotoxicity for fibroblasts and a significant increase of total NO/NO_2_ levels in the cell lysates in vitro and increased levels of 8-OHdG and carbonyl proteins in vivo. Although wound repair in the continuously Ag NPs/CNFS-treated group was delayed, it could be mitigated by washing the covered wound with saline. Thus, Ag NPs/CNFS may become accepted as an anti-infectious wound dressing.

## 1. Introduction

Recently, interest in silver nanoparticles (Ag NPs) and their applications has increased, mainly because of their antimicrobial activities, allowing their use in commercial products such as personal care, household, and medical products as well as in textiles and food products [1,2]. Multiple processes have been reported for controlling the physical and/or chemical characteristics of Ag NPs [3], and environmentally friendly processes have been devised using harmless materials to prepare Ag NPs, precluding the need for complicated purification procedures prior to use in biomedical and environmental applications [4]. We also reported the synthesis of Ag NPs (diameter < 10 nm) using only AgNO_3_-containing glass powder, glucose, and water [4,5,6]. Commercially available AgNO_3_-containing glass is usually used as an antimicrobial agent in environmental, osseous, or dental applications because it allows the sustained release of Ag^+^ into aqueous environments.

Biological and environmental risks of synthetic Ag NPs include adverse effects on some aquatic organisms, including cytotoxicity and genotoxicity in fish [7] and inhibition of photosynthesis in algae [8]. In addition, significant declines in mouse spermatogenic stem cells were observed following treatments with Ag NPs [9]. Several studies have demonstrated that Ag NPs have toxic effects at the cellular, subcellular, and biomolecular levels, such as genes and proteins [2,10]. The evaluation of cytotoxicity of Ag NPs has been carried out in different cellular models [1], including lung fibroblasts [10], glioblastoma cells [10], lung cancer cell line [11], hepatocytes [12], and mesenchymal stem cells [13]. Indeed, oxidative stress and severe lipid peroxidation have been observed in various tissues of fish upon exposure to Ag NPs. The proposed mechanism by which Ag NPs lead to cytotoxicity has been considered to at least partially be through the induction of reactive oxygen species (ROS) [11,14].

Intraperitoneal administration of Ag NPs into mice, at an extremely high concentration (1000 mg/kg), may lead to alterations of gene expression, suggesting neurotoxic effects. Furthermore, there are some reports on oral applications of Ag NPs [15]. After oral exposure, Ag NPs are likely translocated from the gut into the blood stream, inducing inflammation, skin discoloration, and liver damage [16]. Although there is little information available related to pulmonary toxicity of Ag NPs routed by inhalation [16], the oxidative potentials of Ag NPs within the lung may drive the toxicity. Another interesting use of Ag NPs is dermatological applications, such as wound dressings containing Ag NPs. The Ag NPs can have different biological behaviors in wounded skin compared with normal skin, since the liver has been suggested as a secondary target for the toxicity [17,18]. Therefore, methods for preventing the diffusion of Ag NPs into the environment and their uptake into human bodies are necessary before their wide use as an antimicrobial wound dressing [2].

Owing to the biological activity and the safety of chitin/chitosan, these compounds have attracted considerable interest as biomaterial sources for hydrogels, micro/nanoparticles, and membranes and sheets [19]. Chitin/chitosan has been widely studied as a natural cationic biopolymer because of its excellent biocompatibility, biodegradability, nontoxicity [20], antimicrobial [21], tissue adhesive [22], hemostasis [23,24], and wound healing [25,26] properties.

Methods for preventing the diffusion of Ag NPs into the environment and their uptake into the human body are necessary before their wide use as antimicrobial agents [27], since Ag NPs are well known to have toxic effects at the cellular, subcellular, and biomolecular levels. In our previous studies, Ag NPs were stably adsorbed onto chitin/chitosan powders [6,28] and sheets [29] with nanoscale fiber-like surface structures. In those applications, Ag NPs/chitin/chitosan composites showed enhanced stability of Ag NPs and exhibited strong antimicrobial (antiviral, bactericidal, and antifungal) activities. In the previous studies, chitin-nanofiber sheets (CNFS) were used to adsorb and to stabilize Ag NPs, remove the caramel generated during autoclaving, and prevent aggregation and precipitation of Ag NPs. Ag NPs were homogenously dispersed and stably adsorbed onto CNFS with nanoscale fiber-like surface structures [29]. Subsequent studies showed that the bactericidal, antifungal, and antiviral activities of Ag NPs/CNFS increased with Ag NP adsorption, indicating potential applications of CNFS as novel stabilizers and carriers for Ag NPs [27,29]. On the other hand, Ag NPs have exhibited various degrees of in vitro and in vivo cytotoxicity [10]. Moreover, despite the acceptance of chitin/chitosan as a suitable biocompatible carrier agent for Ag NPs [27,29], no study has investigated the cytotoxicity of Ag NPs/CNFS. It is necessary to fully understand the cytotoxicity of Ag NPs/CNFS in dermal applications and its mechanism of action, thereby ensuring its safety in clinical applications as an anti-infectious wound dressing.

We previously reported that Ag NPs were efficiently adsorbed onto CNFS. The resulting Ag NPs/CNFS composites enhanced the stability of Ag NPs and exhibited much stronger antimicrobial activities [27,29]. Although those observations indicate that Ag NPs/CNFS have potential as antimicrobial biomaterials and anti-infectious wound dressings, no further study has been performed on the cytotoxicity and safety of Ag NPs/CNFS for dermatological applications as anti-infectious wound dressing. The aim of this study is to evaluate the cytotoxic effect of Ag NPs/CNFS for fibroblasts in vitro and the inhibitory effect on wound healing of healing-impaired wound in vivo, focused on oxidative stress.

## 2. Results

### 2.1. Cell Proliferation Assay and NO/NO_2_ Measurement In Vitro

**Figure 1:**
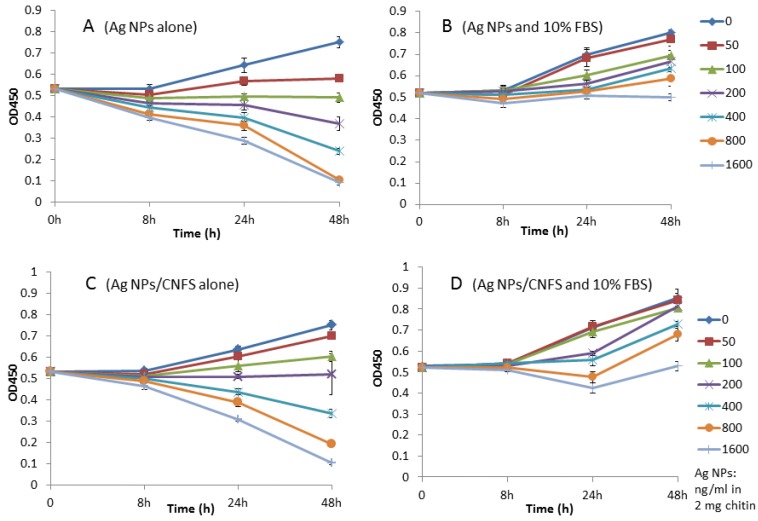
Fibroblast cell proliferation assay. Human fibroblasts were cultured with (**B**,**D**) or without (**A**,**C**) 10% fetal bovine serum (FBS) and various concentrations of silver nanoparticles (Ag NPs) alone or Ag NPs/chitin complex (Ag NPs/CNFS) for 2 days. Data represent the mean ± SD of 6 determinations.

Human fibroblasts were cultured on 24-well tissue culture plates in 1 mL of Dulbecco’s modified Eagle Medium (DMEM) with or without 10% heat-inactivated fetal bovine serum (FBS) and various concentrations of Ag NPs alone or Ag NPs/chitin (2 mg) complex for 2 days to evaluate the cytotoxicity of Ag NPs and Ag NPs/chitin complex [5,6]. Fibroblasts cultured in the presence of ≥100 ng/mL Ag NPs (Figure 1A) or ≥200 ng Ag NPs/chitin (2 mg) (Figure 1C) without FBS showed decreased proliferation during 2-day culture. In contrast, the number of cells increased with 10% FBS in the presence of ≤1.6 μg/mL Ag NPs (Figure 1B) and Ag NPs/chitin (2 mg) (Figure 1D).

**Figure 2:**
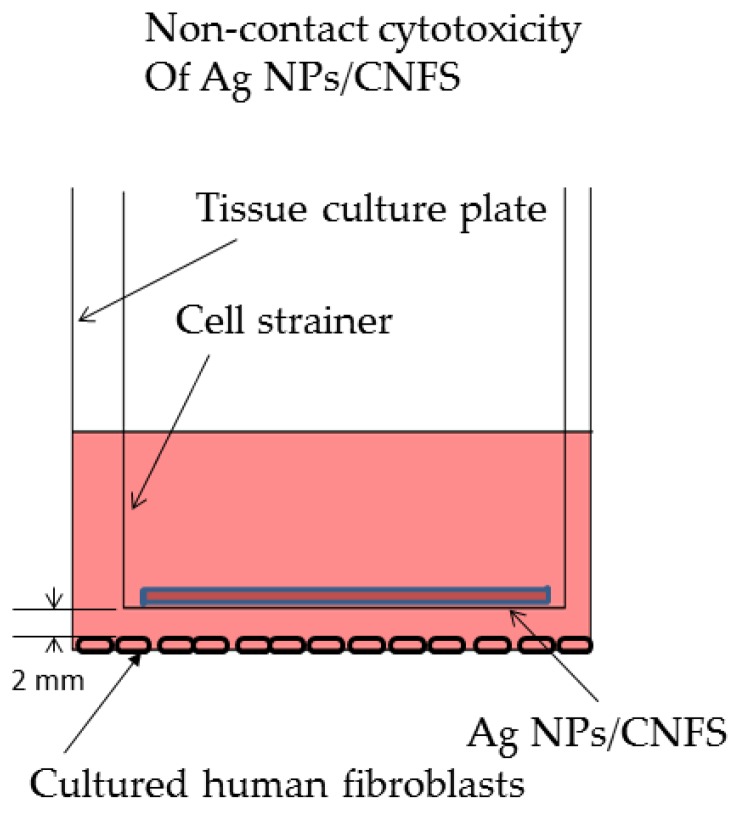
Scheme of experiments on noncontact cytotoxicity of Ag NPs/CNFS (for Figure 3). The fibroblasts were cultured on a 6-well tissue culture plate in 2.5 mL of Dulbecco’s modified Eagle Medium (DMEM) containing 10% FBS for 2 days. Either Ag NPs/CNFS or CNFS alone was placed on a round cell-strainer.

**Figure 3:**
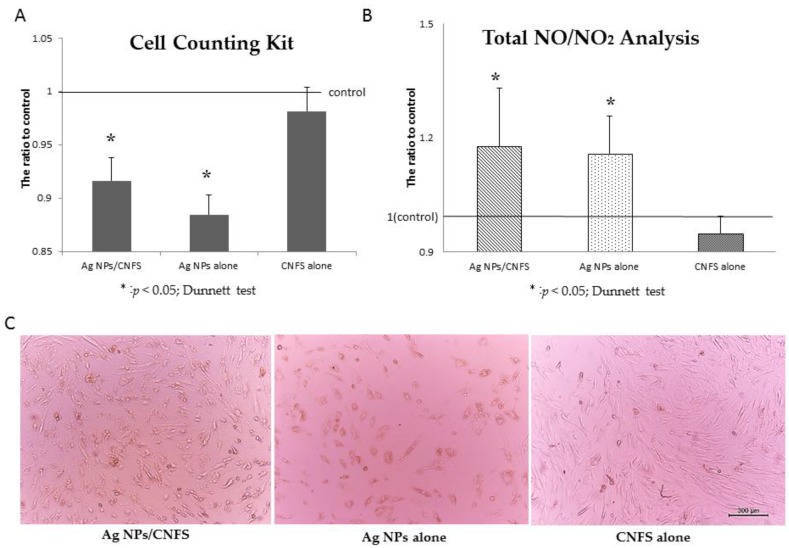
Cytotoxicity of Ag NPs/CNFS in vitro toward fibroblasts. The proliferation of fibroblasts cultured with either Ag NPs/CNFS or Ag NPs as described in Figure 2 decreased for 1-day culture (**A**). Each fibroblast was harvested after cells were exposed to Ag NPs/CNFS for 1 day. The cell lysates were subjected to total NO/NO_2_ analysis (**B**). Data represent the mean ± SD of 6 determinations. Microscopic observation showed that the cells in Ag NPs/CNFS- and Ag NPs-groups were necrotized, and each microphotograph was representative of six cultures (**C**).

The fibroblasts were also cultured on 6-well tissue culture plates in 3 mL of DMEM containing 10% FBS for 2 days. Either Ag NPs (6 μg)/CNFS (diameter 20 mm, about 10 mg) or CNFS alone was placed on a round cell-strainer (diameter: 21 mm). The distance between the cell layer and the cell strainer was 2 mm. For Ag NPs and control groups, 2.5 mL of medium with and without 2 μg/mL Ag NPs, respectively, was poured from the cell strainer (Figure 2). These cultures were incubated in 2.5 mL DMEM with 10% FBS and the antibiotics for 1 day. The cells cultured with either Ag NPs/CNFS or Ag NPs decreased in number compared to control at 1-day culture (Figure 3A). Microscopic observation of Ag NPs/CNFS- and Ag NPs-treated cells showed distinct morphological changes, indicating unhealthy cells and floating cells, and suggested widespread cell death due to necrosis (Figure 3C). In this experiment, there was no direct contact between the cells and Ag NPs/CNFS, and there was no detectable release of Ag NPs into the medium from Ag NPs/CNFS (data not shown). However, the possibility that the cytotoxic effect was caused by Ag^+^-release from Ag NPs/CNFS into medium was not ruled out, since although silver ions (Ag^+^) in the medium were measured using silver ion detection kits, the sensitivity was low. On the other hand, Ag NPs alone permeated through the cell strainer and directly interacted with the cells. Furthermore, total amounts of NO/NO_2_ in the cell lysates of Ag NPs/CNFS (271 ± 70 μmol/mL: ratio = 1.18) and Ag NPs (269 ± 50 μmol/mL: ratio = 1.17) groups were significantly increased compared to the control (230 ± 60 μmol/mL: ratio = 1.0) and CNFS (220 ± 50 μmol/mL: ratio = 0.96) groups (Figure 3B).

### 2.2. Wound Closure and Histochemical Analyses

**Figure 4:**
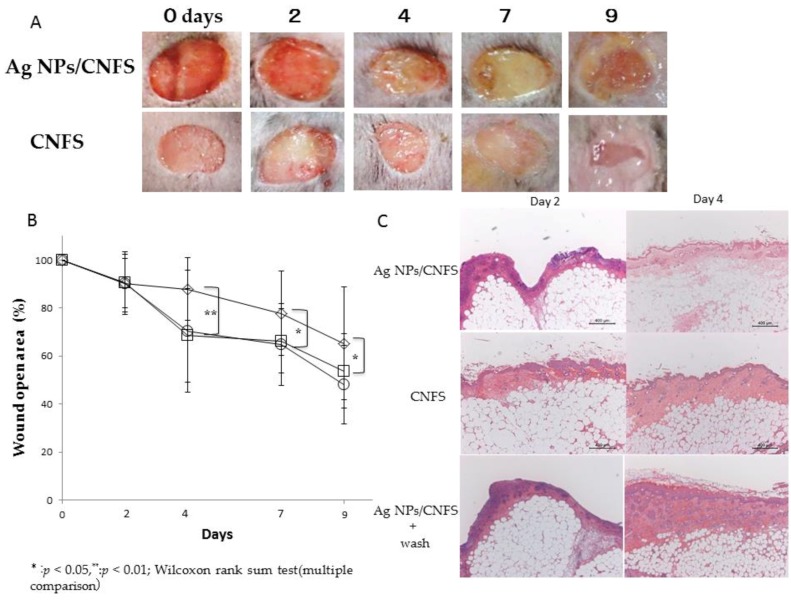
Effects of Ag NPs/CNFS removal and washing on wound epithelialization. Ag NPs/CNFS (◇) and CNFS (○) were removed, and Ag NPs/CNFS was removed and the wound was washed with saline (□) on the indicated day after wound creation, and digital photographs were taken to quantify the epithelialization rates (**A** and **B**). Each photograph was representative of six wounds (**A**). Data represent the mean ± SD of 6 determinations, Wilcoxon rank sum test (multiple comparison), * *p* < 0.05, ** *p* < 0.01 (**B**). Wound closure in the wash group was recovered by removal of Ag NPs/CNFS on day 4–9. Histological examination of reepithelialization and granulation of wounds treated with Ag NPs/CNFS on days 2 and 4, and each microphotograph was representative of six cultures (**C**).

**Figure 5:**
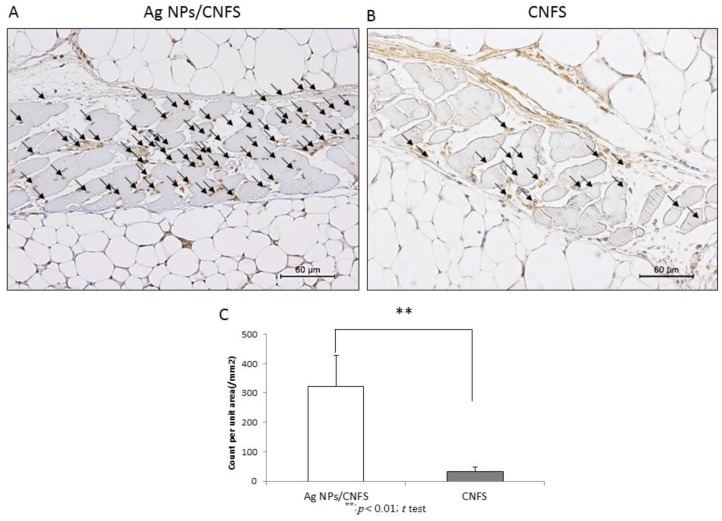
Effect of Ag NPs/CNFS or CNFS treatment on oxidative stress in skin. Immunohistochemical (8-hydroxy-2′-deoxyguanosine, 8-OHdG) staining of skin sections was performed to measure the proportion of 8-OHdG-positive sites 1 day after treatment with either Ag NPs/CNFS (**A**) or CNFS (**B**). A significant increase in the number of 8-OHdG-positive sites was observed in the Ag NPs/CNFS group compared with the CNFS group. The arrows show the 8-OHdG-positive sites. Each microphotograph is representative of six samples (**A**,**B**). The number of 8-OHdG-positive sites in microphotograph of each sample (*n* = 6) were statistically analyzed (**C**). Data represent the mean ± SD of 6 determinations, ** Student’s *t* test, *p* < 0.01 (**C**).

Male well-established diabetic mice (C57BLKS/J Iar-+*Lepr*^db^/+*Lepr*^db^; SLC Japan Inc., Tokyo, Japan) were used (12–16 weeks old) as a healing-impaired wound model. On 2, 4, 7, and 9 days after wounding, each cover sheet was removed and digital photographs were taken to quantify the epithelialization rates. The continuously covered wounds with Ag NPs (6 μg)/CNFS (diameter: 20 mm, about 10 mg) exhibited significantly delayed wound closure on days 4, 7, and 9 compared to wounds covered with CNFS alone (Figure 4A). However, the delay in wound closure was mitigated by washing Ag NPs/CNFS-covered wounds with saline on days 2 and 4 (Figure 4B). Figure 4C shows histological examination of reepithelialization and granulation of wound treated with continuous Ag NPs/CNFS, CNFS alone, and Ag NPs/CNFS with saline wash. The result also indicated mitigation of the delay in wound repair with Ag NPs/CNFS with saline wash. For histological observation, 8-hydroxy-2′-deoxyguanosine (8-OHdG) immunohistochemical staining of skin sections after 24 h was performed to measure the 8-OHdG-positive sites (Figure 5). The 8-OHdG indicates the hyperoxidation state of nucleic acids, allowing evaluation of the antioxidative effect [30]. Significant increases in the number of 8-OHdG-positive sites were observed in the Ag NPs/CNFS-treated wounds compared with the CNFS-treated (control) wounds (Figure 5).

### 2.3. Carbonyl Protein

**Figure 6:**
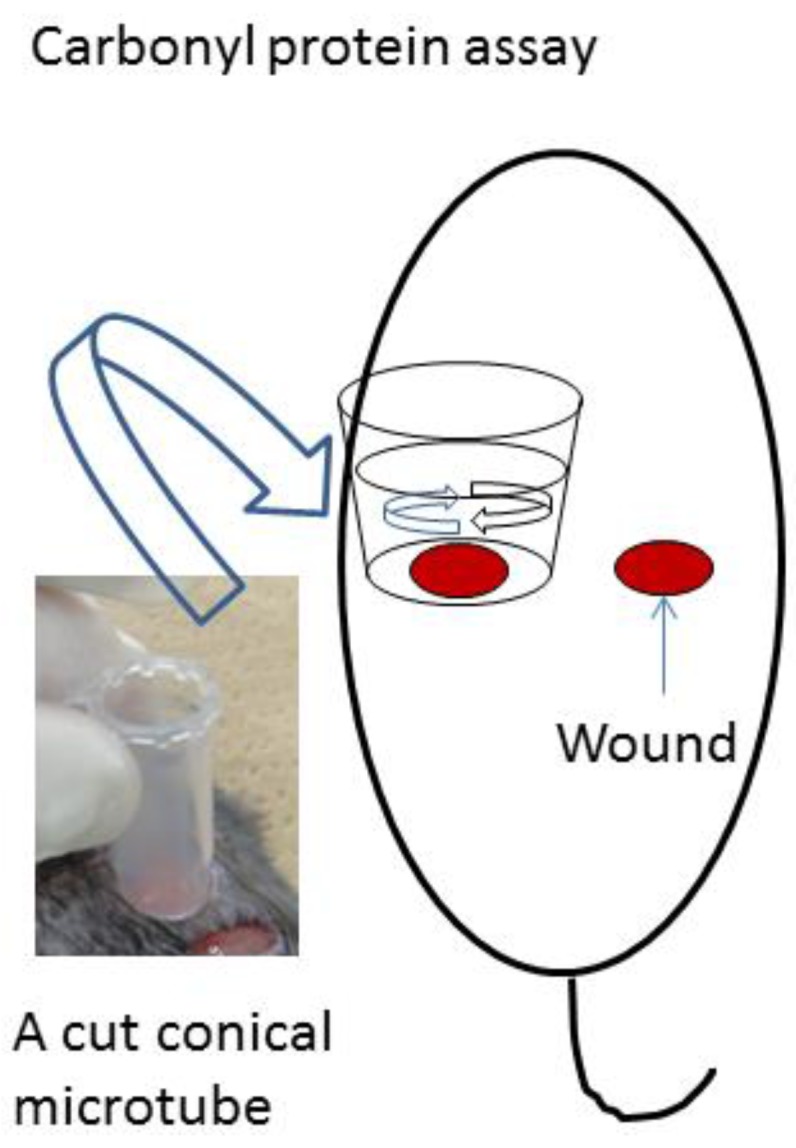
Scheme of experiments on carbonyl protein. A cut conical microtube with phosphate-buffered saline (PBS) was placed on the wound, which was gently washed on days 2, 4, 7, and 9. The washes were collected and measured for the amount of carbonyl protein. The data were analyzed in Figure 7.

**Figure 7:**
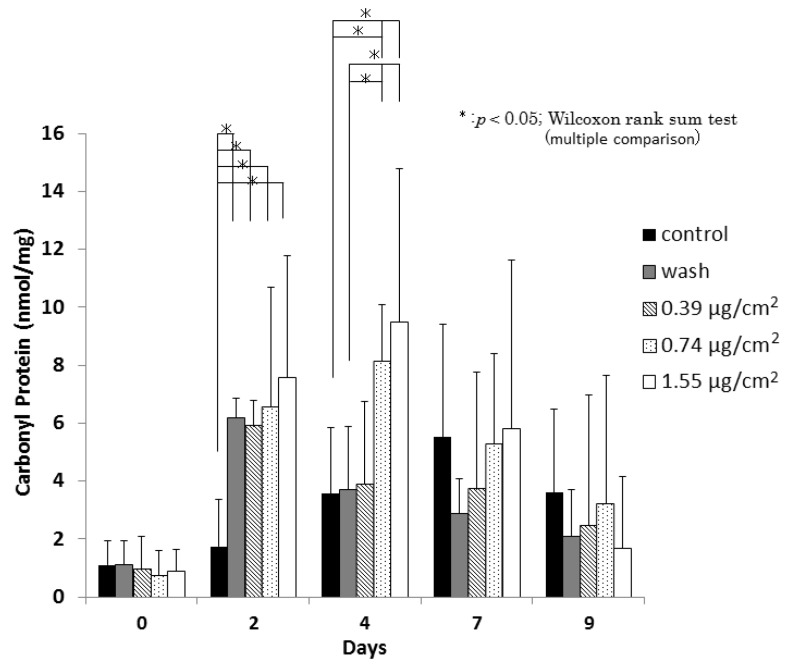
Effect of Ag NPs/CNFS or CNFS treatment on carbonyl protein generation. Carbonyl protein levels in the washes of various concentrations of Ag NPs/CNFS, CNFS, and washed groups were measured, as illustrated in Figure 6. The wash group was treated with 1.55 μg/cm^2^ Ag NPs/CNFS for 2 days and then treated with 1.55 μg/cm^2^ Ag NPs/CNFS after washing the wound with saline on days 2 and 4. Data represent the mean ± SD of 6 determinations, Wilcoxon’s rank sum test (multiple comparison), * *p* < 0.05.

Protein oxidation, which produces carbonyl proteins, is defined as the covalent modification of proteins induced either directly by reactive oxygen species (ROS) or indirectly by reaction with secondary byproducts of oxidative stress [30,31]. Figure 6 shows a scheme of carbonyl protein assay. Carbonyl protein levels in the washes of 0.39 μg/cm^2^, 0.74 μg/cm^2^, and 1.55 μg/cm^2^ Ag NPs/CNFS-treated wounds were significantly higher on day 2 than those in the CNFS-treated group (Figure 7). In contrast, the wash group, which was treated with 1.55 μg/cm^2^ Ag NPs/CNFS for 2 days and then treated with 1.55 μg/cm^2^ Ag NPs/CNFS after washing the wound with saline on days 2 and 4 exhibited significantly lower values on day 4 than those of the continuously 0.74 μg/cm^2^ and 1.55 μg/cm^2^ Ag NPs/CNFS-treated groups. Thereafter, the amounts of carbonyl proteins in each group gradually decreased on day 7 and day 9, and there were no significant differences.

## 3. Discussion

Oxidative stress has specific effects in cells, including oxidative damage to proteins, lipids, and DNA. To establish the role of oxidative stress as a decisive factor in Ag NPs/CNFS toxicity, cell growth assay and measurements of NO/NO_2_ levels were performed in this study. Ag NPs/CNFS as well as Ag NPs alone exhibited cytotoxicity in fibroblasts in this study by direct contact; the cytotoxicity could be partially ameliorated in the presence of 10% FBS (Figure 1). In addition, a significant increase of total NO/NO_2_ levels was also observed in both Ag NPs/CNFS- and Ag NPs-treated cell lysates in vitro. The identical increase of total NO/NO_2_ levels when treated with either Ag NPs/CNFS or Ag NPs might indicate enhanced inflammation [32]. In this study, there was no direct contact between the cells and Ag NPs/CNFS (Figure 3), and there was little detectable release of Ag NPs from Ag NPs/CNFS. However, since it has been reported that Ag^+^ may be rapidly released from Ag NPs in some conditions [33,34,35], the possibility that Ag release from Ag NPs/CNFS into medium was not ruled out. Although silver ions (Ag^+^) in the medium were measured using silver-ion detection kits, the sensitivity was low. Thus, the cellular uptake or direct interaction of Ag^+^ released from Ag NPs/CNFS cannot be ruled out as a major mechanism of the cytotoxicity of Ag NPs/CNFS since toxic concentration of Ag^+^ is very low. However, further investigation is required regarding the possible dissolution and cellular uptake of Ag^+^ or Ag NPs from Ag NPs/CNFS and the long-term safety effects in both humans and for the environment.

Wound healing of *db*/*db* diabetic mice in the Ag NPs/CNFS group in vivo was significantly delayed on days 4, 7, and 9 compared to the CNFS group. However, restoration of wound healing was observed after removing Ag NPs/CNFS and washing the wound with saline (Figure 4). Similarly, while carbonyl proteins obtained from Ag NPs/CNFS-treated wounds were significantly higher on day 2 than the CNFS group, the wash group, which was treated with Ag NPs/CNFS and then treated with Ag NPs/CNFS after washing the wound with saline on days 2 and 4, exhibited significantly lower values on days 4 and 7 (Figure 7). In histological observation, 8-OHdG immunostaining in Ag NPs/CNFS-treated wounds was significantly greater than that observed for CNFS-treated wounds (Figure 5). Carbonyl protein levels in wounds were also significantly higher on day 2 compared to the control (Figure 7). Thus, although Ag NPs/CNFS treatment of wounds results in delayed repair, probably by the generation of oxidative stress, the wound repair can be restored by washing the covered wound with saline on days 2 and 4. Thus, Ag NPs/CNFS may become accepted as an anti-infectious wound dressing.

Several reports have emphasized the role played by oxidative stress in nanoparticle toxicology [10,34,35,36]. To establish the role of oxidative stress as a decisive factor in Ag NPs/CNFS toxicity, measurements of cell proliferation and NO/NO_2_ levels in vitro, and wound repair, 8-OHdG immunostaining, and carbonyl protein levels in vivo were performed. The increase of total NO/NO_2_ levels treated with either Ag NPs/CNFS or Ag NPs might indicate enhanced inflammation as a source of oxidative stress, since NO acts a second messenger in inflammatory signaling and is immediately changed to NO_2_ [32]. The 8-OHdG staining indicates the hyperoxidation state of nucleic acids, allowing the antioxidative effect to be evaluated [30]. The use of carbonyl protein as a biomarker of oxidative stress has some advantages compared to the measurement of other oxidation products, such as the relatively early formation and stability of carbonylated proteins [31]. The results of these oxidative stress biomarkers indicate a close relationship between cytotoxicity and oxidative stress generated from Ag NPs/CNFS. Thus, the proposed mechanism of the cytotoxic effects of Ag NPs is thought to involve, in part, the induction of ROS as oxidative stress.

Finally, CNFS is commercially available, is a clinically approved biomaterial, and is used as a wound dressing. Given the fact that obtaining clinical approval for a wound dressing is time-consuming and costly, to exploit the function and safety of CNFS as Ag NPs-carrying wound dressing seems a more accessible and feasible option. We examined other materials such as cotton, cellulose sheet, collagen sponge, silk, and others on adsorption of Ag NPs. A little Ag NPs are adsorbed to collagen sponge and silk sheet, but Ag NPs are not adsorbed to the others. In addition, a little Ag NPs are adsorbed to chitin/chitosan with flat/smooth film-like surface [6,28]. CNFS has a weak antimicrobial activity and the addition of Ag NPs remarkably enhance microbicidal activity [6,28]. Additionally, chitin/chitosan has been widely studied as a natural cationic biopolymer because of its excellent biocompatibility, biodegradability, nontoxicity, antimicrobial, tissue adhesive, hemostasis, and wound-healing properties [19]. Thus, CNFS is an excellent carrier material for Ag NPs as Ag NPs-carrying wound dressing.

## 4. Materials and Methods

### 4.1. Preparation of Ag NPs and Ag NPs/CNFS

To 100 mL of distilled water containing 1 g d-glucose (Wako Pure Chemical Industries, Ltd., Osaka, Japan), 1 g Ag NO_3_-containing glass powder (BSP21, Kankyo Science, Kyoto, Japan) was added and mixed well. The mixture was autoclaved (121 °C, 200 kPa) for 20 min and then gradually cooled down to room temperature. After centrifugation of the mixture at 1500 rpm for 10 min, the brown supernatant containing Ag NPs was collected and stored at 4 °C.

CNFS (Beschitin W^®^, Unitika Ltd., Osaka, Japan) (70 mm × 55 mm, degree of deacetylation of about 30%) was immersed in 2 mL of various concentrations of Ag NP suspension (particle size 6.53 ± 1.78 nm), washed with distilled water three times, and then dried to produce Ag NPs/CNFS. The concentration of released Ag NPs into the medium from Ag NPs/CNFS was evaluated using UV–Vis spectra, and a peak at 390.5 nm which was representative of the spherical Ag NPs, and silver ions (Ag^+^) were measured using silver-ion detection kits (KDD Corp., Osaka, Japan).

### 4.2. Cell Proliferation Assay and NO/NO_2_ Measurement In Vitro

Human fibroblasts from Takara Bio Inc., Shiga, Japan, manufactured by PromoCell GmbH, Heiderlerg were used in this study. The tissue used by PromoCell for the isolation of human cell cultures was derived from donors who have signed an informed consent form, which outlines in detail the purpose of the donation and the procedure for processing the tissue. The ethical committee in National Defense Medical College does not require approval for usage of commercially available human-derived cells (PromoCell, GmbH, Heidelberg, Germany). The fibroblasts were seeded at a density of about 5 × 10^4^ cells on a 24-well tissue culture plate (Sumitomo Bakelite Co., Ltd., Tokyo, Japan) and cultured in 1 mL of DMEM containing 100 U/mL penicillin G and 100 μg/mL streptomycin with or without 10% heat-inactivated fetal bovine serum (FBS) with various concentrations of Ag NPs alone or Ag NPs/chitin (2 mg) complex for 2 days. The chitin used in this study was prepared by mincing CNFS (diameter > 20 μm). In this experiment, the fibroblasts could grow at almost the same growth rate for 2 days when starved of FBS, probably due to the remaining FBS. The growth rate of fibroblast significantly decreased after 3 days or more compared to that cultured with 10% FBS. Therefore, this experimental protocol was adopted.

The fibroblasts were also seeded at a density of about 5 × 10^4^ cells on a 6-well tissue culture plate (Sumitomo Bakelite Co., Ltd., Tokyo, Japan) and cultured in 3 mL of DMEM containing 10% FBS for 2 days. Ag NPs (6 μg)/CNFS (diameter: 20 mm, about 10 mg), or CNFS alone was placed on a round cell-strainer (40 μm Nylon, BD Falcon, San Jose, CA, USA). The distance between the cell layer and cell strainer was 2 mm (Figure 2). For the Ag NPs group, 2.3 μg of Ag NPs was added into the 2.5 mL of medium poured onto the cell strainer. These cell cultures were incubated in 2.5 mL DMEM with FBS and the antibiotics for 1 day.

To count live fibroblasts, 200 μL WST-1 reagent (Cell Counting Kit, Dojindo, Kumamoto, Japan) was added into the fresh medium (2 mL), incubated for 1 h at 37 °C, and the optical density (OD) was read at 450 nm on an Immuno Mini plate reader (Nunc InterMed Japan, Tokyo, Japan).

For NO/NO_2_ measurement, the strainers and used media were removed and the cells were gently rinsed with phosphate-buffered saline (PBS). The cells were harvested with lysis buffer (Affymetrix Japan K.K, Tokyo, Japan). The lysates were then centrifuged at 1000× *g* for 30 min, and the protein contents in the supernatant were measured using a protein assay kit (Coomassie Protein Assay Kit, Thermo Scientific, Waltham, MA, USA). The protein concentrations were adjusted to 10 μg/mL. The total NO/NO_2_ was measured with a NO/NO_2_ measurement kit (R&D Systems, Minneapolis, MN, USA).

### 4.3. In Vivo Study

All animal experiments were carried out according to the protocol approved by the Animal Experimentation Committee (authorization number: 13014) at the National Defense Medical College (Tokorozawa, Saitama, Japan). The ambient temperature was maintained at 25 ± 2 °C during the experiments. Diabetic mice (C57BLKS/J Iar +*Lepr*^db^/+*Lepr*^db^; SLC Japan Inc, Tokyo, Japan) were used (12–16 weeks old) as a healing-impaired wound model. Before the experiments, urinary glucose and protein in each mouse were analyzed using reagent strips (Uriace, Terumo Corporation, Tokyo, Japan), and all of the *db*/*db* mice were diagnosed as severely diabetic.

Animals were lightly sedated in an ether-filled anesthesia box. After the sedation, intraperitoneal anesthesia was performed. The anesthetic agent was a 5 mg/mL solution of pentobarbital sodium (Somnopentyl^®^, 64.8 mg/mL; Kyoritsu Seiyaku, Tokyo, Japan) in saline, injected at an initial dose of 50 mg/kg body weight. After the hair was shaved using electric clippers, a full-thickness wound with a diameter of 8 mm was created in two places on the back of a mouse by BIOPSY PUNCH (Kai Medical, Tokyo, Japan). The wounds were covered with either Ag NPs/CNFS or CNFS alone, wrapped with film (Kurerap, Kureha Corporation, Tokyo, Japan) and then fixed with an adhesive stretch bandage (HILATE, Iwatsuki Co., Ltd., Tokyo, Japan).

#### 4.3.1. Wound Closure Analyses

On 2, 4, 7, and 9 days after wound creation, the cover sheets were removed and digital photographs were taken to quantify the epithelialization rates. The wound-healing rate was calculated using the following formula: wound area (%) = unepithelialized area/original wound area × 100.

#### 4.3.2. Histochemistry

Excised skin tissues were fixed in 4% paraformaldehyde solution at 4 °C for 12 h, embedded in paraffin, and cut into 3.5 µm wide sections 2 and 4 days after wound preparation. Each section was stained with hematoxylin and eosin (H&E). Sections were made perpendicular to both the anteroposterior axis and the surface of the skin. Each section was stained with H&E.

For staining with 8-hydroxy-2′-deoxyguanosine (8-OHdG), skin sections were deparaffinized, washed with distilled H_2_O, and incubated in 0.3% hydrogen peroxide for 15 min to suppress endogenous peroxidase activity. After washing with PBS, blocking solution was applied to the samples at room temperature for 10 min to adsorb nonspecific proteins. The washed samples were treated with primary antibody (anti-8-OHdG mouse monoclonal antibody, Japan Institute for the Control of Aging, Shizuoka, Japan) for 12 h at 4 °C. The washed samples were treated with secondary antibody (anti-mouse peroxidase-conjugated second antibody, Nichirei Biosciences, Tokyo, Japan) for 30 min at room temperature. After washing the samples, 3-3′-diaminobenzine-4HCl (DAB) (Dako, Glostrup, Denmark) was added and the samples were stained for 5 min. The sections were counterstained with Mayer’s hematoxylin to study tissue morphology.

#### 4.3.3. Measurements of Carbonyl Protein

A cut conical microtube (1.5 mL) with 1 mL of PBS was placed on the wound, which was gently washed on days 2, 4, and 7 as illustrated in Figure 6. The collected washes were then centrifuged (3000 rpm) at 4 °C for 10 min, and the supernatants were subjected to measurement of protein contents using a protein assay kit (Coomassie Protein Assay Kit, Thermo Scientific, Waltham, MA, USA). The protein concentrations were adjusted to 10 μg/mL and carbonyl protein was determined using a protein carbonyl assay kit (OxiselectTM Protein Carbonyl ELISA Kit, Cell Biolabs, Inc., San Diego, CA, USA).

### 4.4. Statistical Analyses

Results were given as mean ± SD. Statistical evaluations were determined using the Wilcoxon rank sum test, Dunnett’s test, or Student’s *t*-test. Statistical analyses were conducted using JMP^®^ Pro for Windows (SAS Institute Inc., Cary, NC, USA. *p*-values of < 0.05 and < 0.01 were considered significant.

## 5. Conclusions

This study suggested that Ag NPs/CNFS has cytotoxicity identical to that of Ag NPs alone. The proposed mechanism of the cytotoxic effects of Ag NPs is thought to involve, in part, the induction of ROS as oxidative stress. Although continuous Ag NPs/CNFS treatment of wounds results in delayed wound repair of healing-impaired diabetic mice, this delay can be mitigated by washing the covered wound with saline. Thus, Ag NPs/CNFS is a promising anti-infectious wound dressing that is applicable for healing-impaired wound repair.

## Abbreviations

Ag NPs/CNFS	silver nanoparticles/chitin-nanofiber sheet composites
8-OHdG	8-hydroxy-2′-deoxyguanosine
NO/NO_2_	nitric oxide/nitrogen dioxide
FBS	heat-inactivated fetal bovine serum
DMEM	Dulbecco’s modified Eagle’s medium
